# ABC Spotlight on single-molecule detection

**DOI:** 10.1007/s00216-020-02838-8

**Published:** 2020-08-27

**Authors:** Günter Gauglitz

**Affiliations:** Institute for Theoretical and Physical Chemistry, Eberhard-Karls-University, Tübingen, Germany

More than 50 years ago, key historical experiments started to detect single molecules using transmission electron microscopy. It began with DNA molecules and proteins, later on came globulin protein molecules in aqueous solution which was more difficult, but allowed measuring the biological molecules in their native state by using fluorescent tags. In the following decades, a large number of methods were developed using super-resolution imaging, near-field scanning microscopy, photo-activated localization microscopy, stimulated depletion microscopy, or super-resolution fluorescence microscopy [1]. Recently, the trends in single-molecule bioanalytical detection with regard to label-based far-field single-molecule imaging, label-free near-field detection of single-molecule interactions, and the suitability for single-molecule clinical assays have been reviewed [2], demonstrating that‚ at these expected very low concentrations, wide-field capturing in label-based single-molecule assays has potential and now it is already used in commercially available kits.

From the beginning of this century, two different approaches regarding the application of single-molecule detection emerged. In one approach, the focus was on monitoring and characterizing a single biological molecule at an interface such as a membrane, measuring cell signaling processes at the membrane or interactions of a single molecule with other molecules. With this approach, the behavior of single molecules in the surroundings or even in cells was in the focus to study dynamics and structure dependence on interactions [3]. Thus, experiments of molecular motors or molecular machines and cell signaling‚ as well as protein dynamics and protein folding‚ were published. As an example, the manipulation of DNA molecules by motor proteins at the single-molecule level is shown [4]. Localization as a tool to restrict effects to extremely small areas created the idea of tailoring nanopores to separate and characterize single molecules. It is considered to be promising to get fundamental knowledge in transport kinetics in glycomics and DNA sequencing [5]. Recently, a nanopore sequencer using protein nanopores has been achieved to sequence a single-molecule base without DNA synthesis or amplification [6]. Furthermore, plasmonic properties of nanoparticles can be used either for information about the interaction of single molecules on these nanoparticles with ligands [7] or allow surface-enhanced Raman spectroscopy [8]. For the characterization of processes in cells, nanoelectrochemistry can also be used, e.g., nanoresolved SECM (scanning electrochemical microscopy). This nanoelectrochemistry can reach high sensitivity, even down to the single-molecule level, and can be used to study single-cell signaling. During brain communication, neurotransmitters are released as chemical messengers. Measuring such transmitters generates key information on neuronal communications and deepens our understanding of brain disorders [9]. There are many fields‚ such as medicine, epidemiology, and ecology‚ that could benefit from this basic research.

Another approach is the determination of very low concentrations in clinical analysis, even down to the single-molecule level. A large number of new plasmonic, waveguide-based or bioelectronic sensors have been developed in the last decade, which target quantification at lower concentrations. This next generation of analytical tools has to face some challenges to achieve quantitative analysis and reduce the limit of detection, as they try to overcome constraints in limits of detection by reducing the detection volume down to the femtoliter***.*** At these very small volumes, there is a restriction and challenge regarding mass transport, i.e., getting enough single molecules into the measured volume within a short time window of the measurement and measuring during the time domain of the interaction. When observing the interaction processes of receptor/ligand sampling at a rather large number of molecules, the reversibility of the binding equilibrium is (according to thermodynamics) not really detectable at low disassociation rate constants. The number of changes in binding events at any time is too small to be detected using classical sensor systems. However, in the case of single-molecule detection, the reversibility might become visible, and the detection method has to become rapid enough to detect the interaction complex [10]. This problem can be overcome by compartmentalization and by a very high loading of recognition elements using advanced immobilization strategies. Diffusion governs the reaction times and can be overcome by introducing electric or magnetic fields to enforce the interaction. As mentioned at the beginning, wide-field capturing arrays with a large number of microreactors or compartments and a large number of recognition elements‚ such as on beads or nanopores‚ can be used to lower concentrations to very small values, even down to the single-molecule detection limit. This can be achieved‚ e.g.‚ by force spectroscopy using an AFM tip [11]. Another possibility is molding of cavities in polydimethylsiloxane for femtoliter arrays, which can allow measurement of such low concentrations. An example is wide-field fluorescence microscopy, which allows detection of interaction processes at this single-enzyme molecule basis in parallel [12]. Another published possibility is single-nanopore potentiostatic recording, which helps to understand the defense mechanisms of bacteria to render antibiotics ineffective [13]. The immobilization of trillions of capturing antibodies to a biosensing platform opens up new relevant opportunities in the field of ultrasensitive point-of-care testing for early diagnosis. This is realized by recognition elements highly loaded to a millimeter-sized gate electrode on a single-molecule transistor platform where the detection of HIV-1 p24 protein at the zM level is recorded [14].

Thus, the development of methods and approaches in the past decades for the detection of single molecules‚ either for a fundamental understanding of the dynamics of the interaction processes or to quantify concentration in clinical assays‚ is a success story. As demonstrated in recent issues of ABC, in the future, new methods and new applications of single-molecule detection will be published in this journal.
